# Sex on the Brain: Reproductive Comorbidities in Temporal Lobe Epilepsy

**DOI:** 10.1177/15357597221135717

**Published:** 2022-11-29

**Authors:** Jenna C. Carpenter, Gabriele Lignani

**Affiliations:** Department of Clinical and Experimental Epilepsy, Queen Square Institute of Neurology, University College London, London

## Abstract

**Increased GABA Transmission to GnRH Neurons After Intrahippocampal Kainic Acid Injection in Mice Is Sex-Specific and Associated With Estrous Cycle Disruption**

Ingram RJ, Leverton LK, Daniels VC, Li J, Christian-Hinman CA. *Neurobiol Dis*. 2022;172:105822. doi:10.1016/j.nbd.2022.105822

Patients with epilepsy develop reproductive endocrine comorbidities at a rate higher than that of the general population. Clinical studies have identified disrupted luteinizing hormone (LH) release patterns in patients of both sexes, suggesting potential epilepsy-associated changes in hypothalamic gonadotropin-releasing hormone (GnRH) neuron function. In previous work, we found that GnRH neuron firing is increased in diestrous females and males in the intrahippocampal kainic acid (IHKA) mouse model of temporal lobe epilepsy. Notably, GABA_A_ receptor activation is depolarizing in adult GnRH neurons. Therefore, here we tested the hypothesis that increased GnRH neuron firing in IHKA mice is associated with increased GABAergic drive to GnRH neurons. When ionotropic glutamate receptors (iGluRs) were blocked to isolate GABAergic postsynaptic currents (PSCs), no differences in PSC frequency were seen between GnRH neurons from control and IHKA diestrous females. In the absence of iGluR blockade, however, GABA PSC frequency was increased in GnRH neurons from IHKA females with disrupted estrous cycles, but not saline-injected controls nor IHKA females without estrous cycle disruption. GABA PSC amplitude was also increased in IHKA females with disrupted estrous cycles. These findings suggest the presence of an iGluR-dependent increase in feed-forward GABAergic transmission to GnRH neurons specific to IHKA females with comorbid cycle disruption. In males, GABA PSC frequency and amplitude were unchanged but PSC duration was reduced. Together, these findings suggest that increased GABA transmission helps drive elevated firing in IHKA females on diestrus and indicate the presence of a sex-specific hypothalamic mechanism underlying reproductive endocrine dysfunction in IHKA mice.

## Commentary

Epilepsy, particularly temporal lobe epilepsy (TLE), is associated with reproductive endocrine disorders in both men and women.^1^ Approximately 60% of women with TLE, not using anti-seizure drugs (ASDs), suffer comorbidities such as menstrual disorders and polycystic ovary syndrome.^2^ In men, semen abnormalities and low serum testosterone levels are common.^1^ Susceptibility to comorbid disturbances is influenced by the location of the epileptic focus, seizure laterality, and the influence of ASDs.^3^ Significantly, the relationship between epileptic activity and reproductive endocrine dysfunction is bidirectional; seizure activity can induce dysfunction of the hypothalamic–pituitary–gonadal (HPG) axis, altering serum levels of gonadal hormones, which can further exacerbate seizure activity.^1^ It is unsurprising, therefore, that the development and impact of reproductive endocrine disorders in epilepsy is significantly impacted by biological sex.

The menstrual cycle, or the equivalent rodent estrous cycle, is controlled by the hypothalamic regulation of gonadotropin secretion from the pituitary gland by gonadotropin-releasing hormone (GnRH) neurons. Gonadotropin-releasing hormone neurons are the central output pathway in the neural control of the HPG axis and are likely mediators of epilepsy-associated reproductive dysfunction.^4^ Previous work in TLE mouse models found altered activity of GnRH neurons to be associated with reproductive disruption in the form of prolonged estrous cycles.^5^ Importantly, estrous stage had a differential effect on GnRH activity in irregularly cycling mice; GnRH neurons were hyperexcitable in diestrus and hypoexcitable, or silent, on estrous. This led the authors to propose that GnRH neuron hyperexcitability in diestrus is a possible driver of comorbid reproductive dysfunction.^5^ Here, Ingram and colleagues aimed to investigate the circuit mechanisms underlying GnRH neuron hyperexcitability.^6^

Ingram and colleagues investigated GABAergic signaling in the hypothalamus as the driver of GnRH neuron hyperexcitability because GABA is the dominant fast synaptic input received by GnRH neurons and paradoxically has an excitatory depolarizing effect in adults similar to the GABA shift seen in immature neurons.^2^ They studied this in the well-established intrahippocampal kainic acid (IHKA) mouse model of TLE. Unilateral IHKA injection results in chronic epilepsy and hippocampal sclerosis following status epilepticus (SE) with histological features of neuronal loss and reactive gliosis.^7^ Status epilepticus was confirmed by video monitoring of behavioral seizures (Racine ≥ 3) in the 5 hours immediately after injection. Chronic epilepsy was not directly established, instead the authors refer to their previous study where 100% of mice were found to develop spontaneous recurrent seizures 1 month after injection.^8^ Cycle monitoring was performed before, and then 1 month after, injection. Importantly, as in humans, not all mice developed reproductive comorbidities, allowing for the neural differences associated with prolonged estrous cycle length to be dissociated from the effects of epilepsy. The female mice were thus stratified into 3 experimental groups: “KA-regular” (4/5 day cycle), “KA-long” (≥7 day cycle), and “saline” controls.

Two months after injection, the authors performed electrophysiological recordings of genetically labeled GnRH neurons in acute hypothalamic slices of mice during diestrus.^6^ The authors observed an increased frequency and amplitude of spontaneous GABAergic inputs onto GnRH neurons in the KA-long group compared to KA-regular and saline groups. The duration of events was also greater for KA-long neurons compared to controls, overall revealing that enhanced GABAergic drive differentiates KA-long and KA-regular mice. Importantly, these differences were not so evident in the presence of blockers of ionotropic glutamatergic transmission. This suggests that hyperexcitability is generated upstream of the GABAergic afferents projecting onto GnRH neurons and that the increased GABAergic drive is potentially due to an increased activity of upstream glutamatergic neurons.^6^

Next, the authors wanted to see whether this effect was also seen in male KA mice. They found no differences in the amplitude or frequency of GABAergic inputs, only observing a slight decrease in event duration.^6^ Reproductive endocrine abnormalities in males were not investigated, which may have drawn out some individual differences. However, testosterone levels were not previously found to be altered for males in this model.^5^

Overall, the authors uncover a sex-specific mechanism for GnRH hyperexcitability in female mice, relating to increased GABAergic drive that is related to estrous cycle disruption.^6^ Their working model is that upstream anteroventral periventricular kisspeptin neurons receive increased glutamatergic inputs which results in a feedforward enhancement of GABAergic transmission onto GnRH neurons. The authors acknowledge, however, that the HPG is regulated by multiple feedback loops and the model they propose may not be the cause of estrous cycle disruption. Future studies could directly test the causal role of increased GABAergic drive by selecting inhibiting the GABAergic neurons and assessing its effect on estrous cycle disruption. Another potential follow-up study could assess the impact of sex hormone feedback on the development of GnRH hyperexcitability. A previous study using the IHKA model found altered levels of E2 and P4 in mice with disrupted cycles, which could influence GABAergic transmission given that these neurons express estrogen receptors, and progesterone metabolites have a powerful antiepileptic effect on the brain.^5^

The important question remains as to why some epileptic mice develop reproductive comorbidities while others do not. The development of reproductive comorbidity does not appear to correlate with seizure burden or the degree of hippocampal sclerosis, suggesting that changes secondary to the primary hippocampal lesion are at play.^9^ In the IHKA model, neuronal loss and circuit reorganization may extend further than the hippocampus, such that it could be worthwhile to perform histological examinations of connected structures. The hippocampus and hypothalamus are synaptically connected, such that the hyperexcitability of the epileptic hippocampus may be driving increased GABAergic signaling in the hypothalamus. Understanding the mechanisms underlying increased GABAergic signaling in vulnerable mice will be important for the development of targeted interventions, especially because some ASDs can further exacerbate endocrine disruption. Whether novel anti-epileptic treatments, such as gene therapy,^10^ targeting hippocampal hyperexcitability can positively impact upon reproductive endocrine comorbidities is an important question, which could shed light on the permanence of such alterations and whether adjunct therapies are needed.

Overall, this study is an important demonstration of sex differences in the development of comorbidities in epilepsy. Stratifying males and females, as well as taking in consideration the estrous cycle stage, can reveal specific mechanisms that may otherwise be masked by variability. The management of reproductive comorbidities through a sex-specific lens is important for seizure control and quality of life, given the potent feedback that peripheral sex hormones can exert on the brain. Research that examines, rather than excludes female variability, promises to deliver more effective treatments.


Jenna C. Carpenter, PhD and Gabriele Lignani, PhD
Department of Clinical and Experimental Epilepsy, Queen Square Institute of Neurology, University College London, London

